# Glass injuries seen in the emergency department of a South African district hospital

**DOI:** 10.4102/phcfm.v7i1.886

**Published:** 2015-09-25

**Authors:** Doudou Nzaumvila, Indiran Govender, Efraim B. Kramer

**Affiliations:** 1 Department of Family Medicine, Sefako Makgatho Health Sciences University, South Africa; 2 Division of Emergency Medicine, School of Medicine, University of the Witwatersrand, South Africa

## Abstract

**Background:**

The emergency department of Embhuleni Hospital frequently manages patients with glass-related injuries. This study assessed these injuries and the glass that caused them in more detail.

**Aim:**

The objectives of our study included determining the type of glass causing these injuries and describing the circumstances associated with different types of glass injuries.

**Setting:**

The emergency department of Embhuleni Hospital in Elukwatini, Mpumalanga province, South Africa.

**Methods:**

This was a cross-sectional study with a sample size of 104 patients. Descriptive statistics were used to assess the characteristics of the glass injuries.

**Results:**

Five different types of glass were reported to have caused the injuries, namely car glass (7.69%), glass ampoules (3.85%), glass bottles (82.69%), glass windows (3.85%) and street glass shards (1.92%). Glass bottle injuries were mainly caused by assaults (90.47%) and most victims were mostly young males (80.23%). The assaults occurred at alcohol-licensed premises in 65.11% of cases. These injuries occurred mostly over weekends (83.72%), between 18:00 and 04:00. The face (34.23%) and the scalp (26.84%) were the sites that were injured most often.

**Conclusion:**

Assault is the most common cause of glass injuries, usually involving young men at alcohol-licensed premises. Glass injuries generally resulted in minor lacerations, with few complications (2.68%).

## Abstract (fr)

**Blessures causées par du verre examinées au service des urgences d’un hôpital de district sud-africain.**

**Contexte:**

Le service des urgences de l’hôpital Embhuleni traite souvent des patients blessés par des éclats de verre. Cette étude a examiné en détail ces blessures et le verre qui les a causées.

**Objectif:**

Notre étude avait pour but de déterminer le type de verre qui avait causé ces blessures et de décrire les circonstances des différents types de blessures causées par du verre.

**Lieu:**

Le service des urgences à l’hôpital Embhuleni à Elukwatini, dans la province du Mpumalanga, en Afrique du Sud.

**Méthodes:**

C’est une étude transversale avec un échantillonnage de 104 patients. On a utilisé des statistiques descriptives pour évaluer les caractéristiques des blessures causées par le verre.

**Résultats:**

Les blessures ont été causées par différents types de verre, à savoir du verre automobile (7.69%), du verre d’ampoules (3.85%), du verre de bouteille (82.69%), du verre de vitre (3.85%) et des éclats de verre dans la rue (1.92%). Les blessures causées par du verre de bouteille provenaient surtout d’agressions (90.47%) et la plupart des victimes étaient des jeunes hommes (80.23%). Les agressions avaient eu lieu dans des locaux autorisés à servir de l’alcool dans 65.11% des cas. Ces blessures ont eu lieu surtout pendant le weekend (83.72%), entre 18h00 et 04h00. Les blessures étaient le plus souvent au visage (34.23%) et au cuir chevelu (26.84%).

**Conclusion:**

Les agressions sont la cause la plus fréquente des blessures causées par du verre et impliquent en général des jeunes hommes dans des locaux autorisés à servir de l’alcool. Les blessures causées par du verre étaient en général des lacérations mineures avec peu de complications (2.68%).

## Introduction

Glass is a material commonly used for glass bottles, cookware and containers. It has the potential to be harmful. Throughout history, glass has been associated with danger, and it is a common cause of injuries warranting visits to the emergency department (ED) of hospitals.^1,2,3,4^ These injuries can be accidental (non-intentional)^5,6^ or intentionally inflicted;^1,2,3^ the latter include ‘glassing’, which refers to alcohol-related violence using glass bottles.^1^

The ED of Embhuleni Hospital in Elukwatini, Mpumalanga province, South Africa, frequently manages patients with glass-related injuries. The severity of wounds varies, with most patients being discharged the same day after sutures and dressing. However, in other patients there may be far worse outcomes, such as the loss of an eye or even death.

Depending on its constitution, glass may be shattered or broken into sharp, jagged pieces or shards which can then cause harm. Glass injuries may occur at home, and this is referred to as a ‘domestic glass injury’ or a ‘home injury’. Domestic injuries or home accidents are a public health problem worldwide.^7^ In 1086 consecutive cases of injuries caused by glass, the home was reported as the most common environment in which they occurred.^8^ Grieshaber and Stegmann reported that glass and other sharp objects represented almost 48% of penetrating eye injuries amongst South African children.^9^ Glass tables are the commonest cause of domestic glass injuries when the victims are children.^10,11^

A ‘street shard glass’ is a piece of broken glass lying on the street. In some studies it was the leading cause of glass injuries for children outside the home.^12^ Children are the main victims of street shard glass injuries; the feet are the most affected body part.^13,14,15^

Many types of worker are at risk of injury due to glass; they include barmen^16,17^ and health workers.^18,19^ Glass ampoules are potentially dangerous if not opened correctly. They account for 26% of needle-less stick and sharps injuries,^20^ which may need microsurgery and extensive rehabilitation, especially when fingers are injured. Needle-less stick injuries can affect non-health workers such as cleaners if glass ampoules and vials are not disposed of in an appropriate sharps container.

Accidental glass injuries are also found amongst victims of road traffic accidents and are usually caused by the windscreen or windshield of a motor vehicle.

Intentional glass injuries are secondary to assault or intentional violence. The incidence and prevalence of violence varies between countries and is mainly associated with alcohol use.^21,22,23^ South Africa has one of the highest rates of interpersonal violence injuries in the world,^24^ and this is associated with alcohol use in from 27% to 47% of cases.^25^ Glass bottles are used impulsively as weapons in alcohol-licensed premises (ALP), in homes or on the street.^24^

Although glass bottles can cause serious injuries, these are rarely fatal; however, such injuries do sometimes need specialist attention. They present mostly located on the scalp and the neck^1,26,27,28^ and when they affect other parts of the body, such as the face and the hands, they may result in unsightly facial scars, disabling hand injuries or other physical deformities, together with post-traumatic stress.^29^ Glass injuries are a common reason for visits to the ED throughout South Africa, but as yet unfortunately little research has been done in the country on this.

The aim of this study was to compile and analyse data on patients with glass injuries presenting to the ED of Embhuleni Hospital.

## Methods

### Design and setting

This was a descriptive prospective study undertaken at Embhuleni Hospital, which is a district hospital with 220 beds in a rural area of Mpumalanga province.

### Sampling and data collection

Data was collected in the ED from 01 February to 31 July 2013 by designated and dedicated ED nurses who were not on duty. There were three nurses who were trained by the researcher; during their training, the aim and objectives of the study and how to collect the data were explained to them. Nurses in the ED were requested to call out one of the trained nurses according to a set roster displayed at the nursing station in order to collect data whenever a patient presented to the ED with a glass injury during the period of study.

Inclusion criteria:

- patients presenting to the ED of Embhuleni Hospital with glass injuries;
- who have consented to participate in the study;
- whose consent has been obtained from a family member or guardian to participate in this study, and
- whose waiver of consent has been obtained to participate in the study.

Exclusion criteria:

- patients presenting to the ED of Embhuleni Hospital with glass injuries;
- who are not under the influence of any recreational drug or alcohol, and
- who have refused to participate in this study.

The following variables were recorded from patients attending the ED:

- Gender, age, address, occupation, highest level of education.
- Persons accompanying the patient to the ED.
- Circumstances of injury.
- Type of glass.
- Type of injury (eg intentional/non-intentional).
- Geographic location of njury occurrence (eg on street, in home).
- Treatment received.
- Single or multiple injuries.
- Characteristics of injury.

Ethical clearance was obtained from Embhuleni Hospital and the ethics committee of the University of the Witwatersrand.

## Results

Amongst the 113 data collection sheets of eligible participants with glass injuries who met the inclusion criteria, 9 were rejected for missing data (3 did not have the type of glass indicated, in 4 the body parts injured were not recorded, and 2 did not indicate the place where the injury occurred).This yielded a final study sample of 104.

Eight participants had injuries from car glass (7.69%), 4 from glass ampoules (3.85%), 86 from glass bottles (82.69%), 4 from glass windows (3.85%) and 2 from street shard glass (1.92%).

**FIGURE 1:**
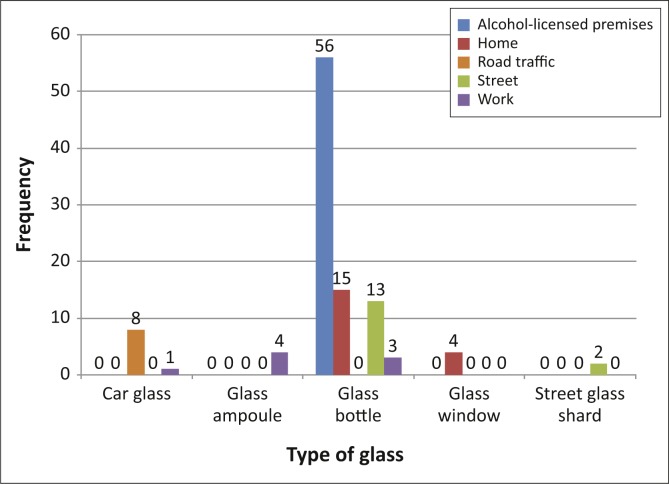
Types of glass and places where the injuries occurred.

Injuries occurred at ALP, in the home, in road traffic accidents, on the street and at workplaces. Injuries in workplaces sometimes overlapped with injuries on ALP and road traffic accidents. Glass ampoules caused injuries at work and on the street. Glass bottle injuries (total: 86 injured patients) as a proportion of all glass injuries occurred as follows: 3 (37.50%) in workplaces (one of the injured patients was a security guard working at an ALP, hence the overlap of one injury whilst at work in an ALP), 15 (78.95%) at home, 13 (86.67%) on the street, and 56 (100.00%) on ALP. Figure 1 indicates the types of glass and places where the injuries occurred.

The minimum age in cases of glass injury was 6 years and the maximum was 57 years. The mean age was 26.04 years. Sixty-seven participants (63.46%) were aged between 15 and 34 years (*p* > 0.10). Glass bottles affected almost all the age groups, whereas injuries from other types of glass were limited to certain age groups. Street shard injuries were found in those of less than 14 years of age (see Table 1).

**TABLE 1:** Demographic characteristics of patients with glass injuries.

Characteristics	Car glass *N* (%)	Glass ampoule	Glass bottle	Glass window	Street shards	Total
**Gender**						
Female	1 (12.50)	4 (100)	17 (19.77)	0	1 (50)	23 (22.10)
Male	7 (87.50)	0	69 (80.23)	4 (100)	1 (50)	81 (77.90)
**Occupation**						
Employed	4 (50)	0	29 (33.72)	2 (50)	0	35 (33.65)
Pupil	2 (25)	0	14 (16.28)	1 (25)	2 (100)	19 (18.27)
Self-employed	2 (25)	0	8 (9.30)	1 (25)	0	11 (10.58)
Student	0	4 (100)	2 (2.32)	0	0	6 (5.77)
Unemployed	0	0	33 (38.37)	0	0	33 (31.73)
**Level of education**						
No formal education	1 (12.50)	0	6 (6.98)	0	0	7 (6.73)
Primary school	1 (12.50)	0	3 (3.49)	1 (25)	2 (100)	7 (6.73)
Secondary school	6 (75)	0	75 (87.20)	3 (75)	0	84 (80.77)
Tertiary school	0	4 (100)	2 (2.32)	0	0	6 (5.77)
**Age groups (yrs)**						
5–9	1 (12.50)	0	0	0	1 (50)	2 (1.92)
10–14	0	0	0	1 (25)	1 (50)	2 (1.92)
15–19	1 (12.50)	0	20 (23.26)	0	0	21 (20.19)
20–24	0	0	26 (30.23)	0	0	26 (25)
25–29	1 (12.50)	2 (50)	15 (17.44)	1 (25)	0	19 (18.27)
30–34	3 (37.50)	1 (25)	16 (18.60)	1 (25)	0	21 (20.19)
35–39	1 (12.50)	0	5 (5.81)	1 (25)	0	7 (6.73)
40–44	0	1 (25)	2 (2.33)	0	0	3 (2.88)
45–49	1 (12.50)	0	1 (1.16)	0	0	1 (0.96)
50–54	0	0	0	0	0	1 (0.96)
55–59	0	0	1 (1.16)	0	0	1 (0.96)

Glass windows affected males only, whereas glass ampoules affected females only. A majority of car glass (7; 87.55%) and glass bottle (69; 80.23%) injuries affected males. All of the unemployed participants (33; 31.73%) were injured by glass bottles.

Fifty-six of the participants (53.85%) were injured in ALP exclusively by glass bottles, as shown in Table 2, which indicates the characteristics of the different types of glass injury.

**TABLE 2:** Characteristics of the different types of glass injury (percentages in parentheses).

Characteristics	Car glass	Glass ampoulampoule	Glass bottle	Glass window	Street shards	Total
**Day of the week**						
Monday	0	0	4 (4.65)	0	0	4 (3.85)
Tuesday	0	0	0	0	0	0
Wednesday	0	0	2 (2.33)	1 (25)	1 (50)	4 (3.85)
Thursday	1 (12.5)	0	7 (8.14)	0	1 (50)	9 (8.65)
Friday	3 (37.5)	0	11 (12.79)	1 (25)	0	15 (14.42)
Saturday	1 (12.5)	0	37 (43.02)	1 (25)	0	39 (37.5)
Sunday	3 (37.5)	4 (100)	25 (29.07)	1 (25)	0	33 (31.73)
**Estimated time when injury happened**						
08:00–12:59	0	2 (50)	5 (5.81)	1 (25)	0	8 (7.69)
13:00–17:59	1 (12.5)	1 (25)	8 (9.30)	0	1 (50)	11 (10.58)
18:00–22:59	3 (37.5)	1 (25)	22 (25.58)	3 (75)	1 (50)	30 (28.85)
23:00–03:59	2 (25)	0	42 (48.84)	0	0	44 (42.31)
04:00–07:59	2 (25)	0	9 (10.47)	0	0	11 (10.58)
**Location where the injuries occurred**						
ALP	0	0	56	0	0	56 (52.92)
Home	0	0	15 (65.12)	4 (100)	0	19 (17.92)
Road traffic	8 (87.5)	0	0	0	0	8 (7.55)
Street	0	0	13 (15.12)	0	2 (100)	15 (14.15)
Work	1 (12.5)	4 (100)	3 (2.32)	0	0	8 (7.55)
**Escort**						
EMS	7 (87.5)	0	50 (58.14)	0	2 (100)	59 (56.73)
Personnel family	1 (12.5)	0	13 (15.12)	4 (100)	0	18 (17.31)
member only	0	0	6 (6.98)	0	0	6 (5.77)
Friends only	0	4 (100)		0	0	13 (12.5)
None	0	0	9 (10.46)	0	0	8 (7.69)
SAPS officer			8 (9.30)			
**Number of injuries**						
Single	4 (50)	4 (100)	47 (54.65)	4 (100)	2 (100)	61 (58.65)
Multiple	4 (50)	0	39(45.35)	0	0	43 (41.35)
**Injured body parts**						
Abdomen	0	0	1 (1.00)	0	0	1 (1)
Arm	0	0	6 (4.80)	0	0	6 (4.03)
Back	2 (14.28)	0	0	0	0	2 (1.34)
Chest	0	0	14 (11.20)	0	0	14 (9.39)
Face	9 (64.28)	0	42 (33.60)	0	0	51 (34.23)
Foot	0	0	0	0	2 (100)	2 (1.34)
Forearm	1 (7.14)	0	12 (9.60)	1 (25)	0	14 (9.39)
Hand	0	4 (100)	10 (8.00)	3 (75)	0	17 (11.41)
Leg	1	0	0	0	0	1 (0.67)
Neck	0	0	2 (1.60)	0	0	2 (1.34)
Scalp	2 (14.28)	0	38 (30.40)	0	0	40 (26.84)

ALP, alcohol-licensed premises; EMS, emergency medical services.

Of the 19 (16.27%) participants injured at home, glass bottles caused injury to 15 (78.95%) and the remaining 4 (21.05%) were injured by glass windows. The 8 (7.69%) occupational glass injuries were caused by car glass, glass ampoules or glass bottles. Of the 15 (14.42%) participants injured on the street, 13 (86.67%) were injured by glass bottles and 2 (13.33%) by street shard glass.

There was a trend of increasing numbers of injuries as the week progressed from Monday, with 87 (84%) occurring over the weekends, 39 of them (37.5%) on Saturdays alone. There was also an increasing number of injuries from 17:00, with more than 74 (70%) of the glass injuries occurring between 18:00 and 04:00.

Intentional glass injuries were inflicted with glass bottles. Amongst the 86 glass bottle injuries, 80 (93%) were from assaults with glass bottles; 51 (63.75%) of the victims laid criminal charges. Fifty-nine participants (56.73%) arrived escorted by emergency medical services (EMS) personnel, whereas 18 (17.31%) were accompanied by relatives, and South African Police Service (SAPS) officers escorted 8 (7.69%). Overlaps were noted, especially between EMS and relatives as escorts.

The minimum length of an injury was 0.5 cm (121 injuries; 81.2%) and the maximum length of an injury was 16 cm. The mean length of the injuries was 3.67 cm.

Of the 149 glass injuries, 118 (79.14%) were intentional injuries, of which 71 (60.17%) were inflicted on the left side of the body. The face was the most common area involved, with 51 injuries (34.23%), followed by the injuries to the scalp (40; 26.84%).

Most patients (94; 90.38%) were discharged after treatment in the ED. The outcomes in all of the cases of glass injuries are indicated in Table 3.

**TABLE 3:** Outcome of the glass injuries (percentages given in brackets).

**Characteristics**	**Car glass**	**Glass ampoule**	**Glass bottle**	**Glass window**	**Street shards**	**Total**
**Outcome**						
Discharged after treatment in ED	8 (100)	3 (75)	77 (89.53)	0	2 (100)	94 (90.38)
Admitted in short- stay room in ED	0	0	6 (6.98)	0	0	6 (5.77)
Transferred	0	1 (25)	3 (3.49)	-	0	4 (3.85)
**Complications**						
Anxiety disorder	0	1 (25)	0	0	0	1 (0.96)
Disembowelment	0	0	1 (1.16)	0	0	1 (0.96)
Eyeball perforation	0	0	1 (1.16)	0	0	1 (0.96)
Tendon injury	0	0	1 (1.16)	0	0	1 (0.96)

ED, emergency department.

The minimum total cost of managing one glass injury patient using the International Classification of Diseases (ICD 10) is estimated to be R1267.47.

There were 86 glass bottle injuries, which represented 82.69% of all glass injuries, with the majority occurring amongst male participants (80.23%) with a minimum age of 16 years and maximum age of 57 years (*p* = 0.0426) (Table 4); 26 (30.23%) of them were young adults aged between 20 and 24 years. It was also found that 3 (3.49%) were minors. It seems that age is significantly related to glass bottle injuries. Of those injured by glass bottle, 33 (38.37%) were unemployed and 75 (87.21%) had a secondary school level of education.

Amongst the 86 participants with glass bottle injuries, the majority of the injuries were due to assault (93.02%), and 56 (65.11%) reported that they were intentionally injured at ALP. Of the 3 minor participants injured by glass bottles, 2 (3.57%) were injured at ALP.

**TABLE 4:** Demographic characteristics of participants with glass bottle injuries.

Characteristics	Frequency	Percentage
**Gender**		
Female	17	19.77
Male	69	80.23
**Occupation**		
Employed	29	33.78
Pupil	14	16.28
Self-employed	8	9.30
Student	2	2.33
Unemployed	33	38.37
**Level of education**		
No formal education	6	6.98
Primary school	3	3.49
Secondary school	75	87.21
Tertiary school	2	2.33
**Age groups (yrs)**		
15–19	20	23.26
20–24	26	30.23
25–29	15	17.44
30–34	16	18.61
35–39	5	5.81
40–44	2	2.33
45–49	1	1.16
50–54	0	0
55–59	1	1.16

A majority of glass bottle injuries (73; 84.88%) occurred over the weekend, and there was an increasing trend towards Saturday (37; 43.02%). The glass bottle injuries were intentional in 76 (90.47%) of cases and were single injuries in 46 (54.76%) (*p* ≤ 0.0019) when comparing injury and glass bottle injuries.

Out of a total of 125 glass bottle injuries, 42 (33.60%) were on the face and 38 (30.40%) on the scalp. Thus the face and the scalp were affected in almost two-thirds of glass bottle injuries.

## Discussion

Glass bottles were responsible for most injuries (82.69%). Laing, Sendall and Barker also found the most common cause of glass injuries (in 75% of their patients) to be glass bottles.^1^ When considering the location of the injuries, our study differed from others because although the glass bottle remained the leading cause of injuries, the percentage significantly differed from 75% in general to 45% in ALP, where other types of glass such as drinking glasses accounted for 44% of glass injuries.^1^ This difference is due to the circumstances found in South Africa. The first factor has to do with local habits and customs in ALP, where beer is not served in drinking glasses but in glass bottles.^30^ The second factor is that the study site is a rural area, where some glass objects are considered to be luxuries (such as glass doors, glass tables) and most of the people cannot afford these.

Male participants made up 77.90% of the participants. This is consistent with other international studies, which found that more males than females are injured with glass. A study of 1086 consecutive injuries caused by glass found a 7:3 male to female ratio,^8^ and another study found that 72% of injuries affected males.^1^ Some authors attributed the large proportion of glass bottle injuries in males to the consumption of alcohol, and thereafter greater risk-taking behaviour.^1^ Other studies confirmed the relationship between the consumption of alcohol by males and violence using glass bottles at ALP.^30,31^

Injuries with glass ampoules were found amongst female student nurses only, which is similar to Smith's findings amongst nursing students.^20^ This is because it is nurses who usually break glass ampoules in order to administer the contents.

Although glass injuries can affect any age group, some are more prone to certain types of glass injury, especially those inflicted by glass bottles and street shard glass.^32^ Other studies also found the age group 20–24 years to have the highest number of glass injuries.^1^ Youth violence, particularly amongst males, is exceptionally high in South Africa,^24^ and our study had similar results, with intentional injuries found to be associated with glass bottles in 80 (93.02%) cases.

Street shard glass injuries (2%) in children walking barefoot were consistent with previous studies that focused on glass injuries in children, which could explain the higher percentage in these studies.^13,33,34^

Occupational glass injuries accounted for 7.55% of all injuries. Occupational injuries involved glass bottles, car glass and glass ampoules. Glass ampoule injuries accounted for 4% of glass injuries and affected the hands, which is common in this type of injury.^20^ The low percentage in this study does not accurately reflect the reality of glass ampoule-related injuries at Embhuleni Hospital, since it is most likely that many health workers, especially nurses, treat these as minor injuries without any reporting or medical consultation. These underreported injuries were also highlighted by Milner,^35^ who found that up to 70% of health workers failed to report them. Multiple injuries were associated with car glass (50%) compared to the other glass types, which resulted only in single injuries. Glass bottles are mainly associated with assault, causing multiple lacerations.^1,36,37^ Car glass has multiple fragments, which cause multiple lesions that are minor unless they affect the eyes or become retained as foreign bodies due to their small size.^38,39^

The windscreens of cars caused 9% of glass injuries.^14^ The face was the most affected body part (9; 64%), which is similar to what has been found in other studies.^40,41,42^

A glass bottle can be used as a dangerous weapon, and commonly affects the head in intentional injuries.^1,2,3,26,27,43^ In our study it was found that glass bottles caused 84% of injuries and affected various body parts. The head was affected in 61.07% of glass bottle injuries. Other body parts such as the hands and forearms were also injured, but less frequently than the head and face – the hands and forearms are used by the victim as a defence mechanism. These findings are similar to those of other research regarding body parts affected by glass bottle injuries.^1,29^

Glass windows are the commonest cause of hand injuries.^5,6,16^ Of the participants with this type of injury, 75% and 25% of them were injured on the hand and forearm respectively. These findings are in line with other studies.^44,45^

The foot was the only body part affected by street shard glass, as reported by other studies.^35,36,37^

There was a trend of increased glass injuries over weekends; this finding is consistent with other international and local studies which have focused on alcohol-related injuries presenting to the ED.^1,22,23,43^ Intentional glass injuries were found only to have been caused by glass bottles and represented 93.02% of participants. These were used in interpersonal violence, which takes place amongst 20% – 47% of alcohol consumers in South Africa.^24^ These glass bottle injuries are consistent with what is known about glass injuries,^1,2,25^ but in our study all injuries occurred at places where alcohol was sold. In this respect our study differed from that conducted by some authors, who found that glass bottle assaults occurred commonly at home.^1^ The pattern of days on which glass bottle injuries occurred is the same as for alcohol consumption in South Africa.^43^ It was reported by some authors that alcohol consumption amongst the youth is a major contributor to crime, violence and intentional and unintentional injuries, as well as other social, health and economic problems.^43^ Thus it is not unexpected that glass bottle injuries occurred during the weekend and were associated with violence.

These assaults with glass bottles were reported to the police in 61% of cases. This is in contrast to other countries, where such assaults with minor lesions and complications, as found in this study, are generally underreported.^31^

Most of these patients had minor injuries but used ambulances as transport to the hospital. Since Embhuleni Hospital is situated in a rural area, members of these poor communities do not have private transport or cannot afford transport to the hospital, so they use the ambulance as a means of accessing medical services, irrespective of whether or not it is an emergency. These injuries occurred mainly at night, when there is no public transport available, and the ambulance is their only means of travelling to hospital. Also, the most commonly injured body parts were the scalp and the face (61.07%); clinics in the area do not suture lacerations of the face and scalp, so these patients are referred to the hospital.

Factors that can explain the choice of a glass bottle in interpersonal violence are its being the only weapon available at the time, the shape of the bottle and the value of the bottle. In 86% of injuries resulting from interpersonal violence with glass bottles, these occurred on ALP. The majority of beer bottles are light, and consist of a body and a neck which is as long as any knife handle. This neck enables a person to secure an easy and comfortable grasp around it and to throw it at or strike a person with force, so that the glass bottle breaks on impact or stabs a person when broken with minimal force.^1,26,27^

The gender of the victim must also be considered as a social impact. In this study, of the 76 intentional glass bottle injuries, 59 (77.63%) were in males injured by the assailant using a glass bottle as a weapon. Although a glass bottle can be considered to be a weapon used on impulse, it causes injuries which are similar to those caused by any sharp object.^46^ Glass bottles are used to take advantage of and neutralise an opponent.^30,47^

All the unemployed participants in this study were injured by glass bottles as a result of interpersonal violence, with 61% taking place on ALP. There seems to be a relationship between glass bottle injuries and unemployment: the high level of unemployment amongst young people in South Africa is a contributing factor to the high rate of liquor consumption, as alcohol is used as a means of relieving stress and is easily purchased from different types of premises.^43,48^

The age distribution for glass bottle injuries showed that the mean is 25.81 years, which is supported by the results of a similar previous study.^49^ This represents the economically active age group. Unfortunately most of them were unemployed. In this age group it was found that due to their negative socio-economic situation their alcohol consumption is high^36,49^ and they are prone to interpersonal violence.^48^ Unemployment, poverty and abuse of alcohol are linked, and these factors are known to be generators of violence.^36,43^

## Limitations

The study period was from 01 February to 31 July 2013, and the injuries over the holiday and/or Christmas period may be different due to availability of more free time and disposable income resulting from annual financial bonuses.

## Conclusion

Victims of glass bottle injuries are usually young unemployed males who were injured on ALP. The injuries from assaults were mostly located on the left side of the body. The face was injured the most by car glass and glass bottle injuries, whereas the feet and hands were mostly affected by street shards and glass ampoules respectively. Glass bottle injuries were associated with assault, and most of these cases were reported to the police. The patients injured by glass mostly used ambulances to attend the hospital ED. Glass recycling can be used at or near ALP to generate income and prevent many glass injuries.

## References

[1] Laing AJ, Sendall MC, Barker R. Alcohol-related violence presenting to the emergency department: Is ‘glassing’ the big issue? Emerg Med Australasia. 2013;25(6):550–557. PMID: 24118859, http://dx.doi.org/10.1111/1742-6723.12136 10.1111/1742-6723.12136 24118859

[2] Emby DJ. Retained glass fragments in body tissues. S Afr Med J. 2009;99(12):858–859. PMID: 20459988, http://dx.doi.org/10.7196/samj.3570 20459988

[3] Nolan G, Lawes S, Hainsworth S, Ruuty G. A study considering the force required for broken glass bottles to penetrate a skin simulant. Int J Legal Med. 2011;126(1):19–25. PMID: 21336639, http://dx.doi.org/10.1007/s00414-011-0556-7 21336639 10.1007/s00414-011-0556-7

[4] Levine MR, Gorman SM, Yarnold PR. A model for teaching bedside detection of glass in wounds. Emerg Med J. 2007;24(6):413–416. PMID: 17513538, http://dx.doi.org/10.1136/emj.2007.047340 17513538 10.1136/emj.2007.047340 PMC2658276

[5] Rothschild MA, Karger B, Schneider V. Puncture wounds caused by glass mistaken for stab wounds with a knife. Forensic Sci Int. 2001;121(3):161–165. PMID: 11566419, http://dx.doi.org/10.1016/S0379-0738(01)00392-9 11566419 10.1016/s0379-0738(01)00392-9

[6] Karger BR, Rothschild MA, Pfeiffer H. Accidental sharp force fatalities – Beware of architectural glass, not knives. Forensic Sci Int. 2001;123(2–3):135–139. PMID: 11728738, http://dx.doi.org/10.1016/S0379-0738(01)00526-6 11728738 10.1016/s0379-0738(01)00526-6

[7] Majori S, Ricci G, Capretta F, Rocca G, Baldovin T, Buinocore F. Epidemiology of domestic injuries. A survey in an emergency department in north-east Italy. J Prev Med Hyg. 2009;50(3):164–169. PMID: 20411650. 20411650

[8] Ousby J, Wilson DH. 1086 Consecutive injuries caused by glass. Injury. 1982;13(5):427–430. PMID: 7085060, http://dx.doi.org/10.1016/0020-1383(82)90099-7 7085060 10.1016/0020-1383(82)90099-7

[9] Glassforeurope [Internet] Main types of glass. [updated 2013 May 23; cited 2014 Aug 17]. Available from: http://www.glassforeurope.com/en/products/main-types-of-glass.php

[10] Kupelian AS, Tincello DG. Penetrating glass injury to the perineum. J Obstet Gynaecol. 2004;24(6):713–714. PMID: 16147628, http://dx.doi.org/10.1080/01443610400008230 16147628 10.1080/01443610400008230

[11] Kimia AA, Waltzman ML, Shannon MW, et al. et al. Glass table-related injuries in children. Pediatr Emerg Care. 2009;25(3):145–149. PMID: 19262417, http://dx.doi.org/10.1097/PEC.0b013e31819b41c8 19262417 10.1097/PEC.0b013e31819b41c8

[12] Al-Khatib IA. Children's perceptions and behavior with respect to glass littering in developing countries: A case study in Palestine's Nablus district. Waste Manag. 2009;29(4):1434–1437. PMID: 19019670, http://dx.doi.org/10.1016/j.wasman.2008.08.026 19019670 10.1016/j.wasman.2008.08.026

[13] Makary MA. Reported incidence of injuries caused by street glass among urban children in Philadelphia. Inj Prev. 1998;4(2):148–149. PMID: 9666372, http://dx.doi.org/10.1136/ip.4.2.148 9666372 10.1136/ip.4.2.148 PMC1730353

[14] The glass recycling company [Internet] Why recycle [cited 2014 Aug 27] Available from: http://www.theglassrecyclingcompany.co.za/index.php?option=com_content&view=article&id=216&Itemid=52

[15] Northglen News [Internet] Glass recycling increased [updated 2013 Mar 20; cited 2014 Aug 27]. Available from: http://www.looklocal.co.za/looklocal/content/en/north-durban-and-umhlanga/north-durban-and-umhlanga-news-general?oid=7129321&sn=detail&pid=1171318&glass-recycling-increases

[16] Evans DM. Hand injuries due to glass. J Hand Surg. 1987;123(5):284 PMID: 3624998. 10.1016/0266-7681_87_90035-0 3624998

[17] McLean W, Shepherd JP, Brann CR, Westmoreland D. Risks associated with occupational glass injury in bar staff with special consideration of Hepatitis B infection. Occup Med. 1997;47(3):147–150. PMID: 9156469, http://dx.doi.org/10.1093/occmed/47.3.147 10.1093/occmed/47.3.147 9156469

[18] Guo YL, Shiao J, Chuang YC, Huang KY. Needlestick and sharps injuries among health-care workers in Taiwan. Epidemiol Infect. 1999;122(2):259–265. PMID: 10355790. 10355790 10.1017/s0950268899002186 PMC2809614

[19] Jagger J, Deitchman S. Hazards of glass capillary tubes to healthcare workers. JAMA. 1998;280(1):31 PMID: 9660356, http://dx.doi.org/10-1001/pubs.JAMA-ISSN-0098-7484-280-1-jbk0701 9660356 10.1001/jama.280.1.31

[20] Smith DR, Leggat PA. Needlestick and sharps injuries among nursing students. J Adv Nurs. 2005;51(5):449–455. PMID: 16098161, http://dx.doi.org/10.1111/j.1365-2648.2005.03526.x 16098161 10.1111/j.1365-2648.2005.03526.x

[21] Humphrey G, Casswell S, Han DY. Alcohol and injury among attendees at a New Zealand emergency department. NZ Med J. 2003;116(1168):1–10. PMID: 12601422. 12601422

[22] Tjipto AC, Taylor DM, Liew H. Alcohol use among young adults presenting to the emergency department. Emerg Med Australasia. 2006;18(2):125–130. PMID: 16669937, http://dx.doi.org/10.1111/j.1742-6723.2006.00819.x 10.1111/j.1742-6723.2006.00819.x 16669937

[23] Sivarajasingam V, Morgan P, Shepherd J, Matthews K. Vulnerability to assault injury: An emergency department perspective. Emerg Med J. 2009; 26(10):711–714. PMID: 19773489, http://dx.doi.org/10.1136/emj.2008.061580 19773489 10.1136/emj.2008.061580

[24] Norman R, Schneider M, Bradshaw D, et al. Interpersonal violence: An important risk factor for disease and injury in South Africa. Popul Health Metr. 2010;8:32 PMID: 21118578, http://dx.doi.org/10.1186/1478-7954-8-32 21118578 10.1186/1478-7954-8-32 PMC3009696

[25] Norman R, Bradshaw D, Schneider M, et al. Estimating the burden of disease attributable to interpersonal violence in South Africa in 2000. S Afr Med J. 2007;97(8):653–656. PMID: 17957838. 17957838

[26] Madea B, Schmidt P, Lignitz E, Padosch SA. Skull injuries caused by blows with glass bottles. Forensic Path Rev. 2005;2:27–41. http://dx.doi.org/10.1385/1-59259-872-2:027

[27] Madea B, Lignitz E. A response to “S.A. Bolliger, S. Ross, L. Oesterhelweg, M.J. Thali, B.P. Kneubuehl, Are full or empty beer bottles sturdier and does their fracture-threshold suffice to break the human skull?”[J Forensic Leg Med 2009 16 138–142]. J Forensic Leg Med. 2009;16(7):432 PMID: 19733339, http://dx.doi.org/10.1016/j.jflm.2009.04.001 19239964 10.1016/j.jflm.2008.07.013

[28] Bolliger SA, Ross S, Oesterhelweg L, Thali MJ, Kneubuehl BP. Are full or empty beer bottles sturdier and does their fracture-threshold suffice to break the human skull? J Forensic Leg Med. 2009;16(3):138–142. PMID: 19239964, http://dx.doi.org/10.1016/j.jflm.2008.07.013 19239964 10.1016/j.jflm.2008.07.013

[29] Magennis P, Shepherd JP, Hutchison I, Brown A. Trends in facial injury. BMJ. 1998;316(7128):325–326. PMID: 9487161, http://dx.doi.org/10.1136/bmj.316.7128.325a 9487161 10.1136/bmj.316.7128.325a PMC2665530

[30] Shepherd JP. The circumstances and prevention of bar-glass injury. Addiction. 1998;93(1):5–7. PMID: 9624706. 9624706 10.1080/09652149836179

[31] Hocking MA. Assaults in southeast London. J R Soc Med. 1989;82(5):281–284. PMID: 2754682. 2754682 10.1177/014107688908200512 PMC1292133

[32] Anderson ER, Grant HI, Weissman M. Penetrating glass injury to the sacral spine. Clin Med Res. 2010;8(2):114–115. PMID: 20660937, http://dx.doi.org/10.3121/cmr.2010.927 20660937 10.3121/cmr.2010.927 PMC2910099

[33] Baker MD, Selbst SM, Lanuti M. Lacerations in urban children. A prospective 12-month study. Am J Dis Child. 1990;144(1):87–92. PMID: 2294725. 2294725 10.1001/archpedi.1990.02150250097042

[34] Armstrong AM, Molyneux E. Glass injuries to children. BMJ. 1992;304(2):360 PMID: 1540735. 1540735 10.1136/bmj.304.6823.360 PMC1881214

[35] Milner A. Needleless intravascular access. South Afr J Anaesth Analges. 2005;11(3):97–101. http://dx.doi.org/10.1080/22201173.2005.10872407

[36] Popovici I, French MT. Does unemployment lead to greater alcohol consumption? Ind Relat. 2013;52(2):444–466. PMID: 23543880, http://dx.doi.org/10.1111/irel.12019 10.1111/irel.12019 PMC3609661 23543880

[37] Shepherd JP, Shapland M, Pearce NX, Scully C. Pattern, severity and aetiology of injuries in victims of assault. J R Soc Med. 1990;83(2):75–89. PMID: 2319550, http://dx.doi.org/10.1177/014107689008300206 2319550 10.1177/014107689008300206 PMC1292500

[38] Safeglass [Internet] Glass history – An overview [updated 2007; cited 2014 Aug 27]. Available from: http://www.breakglass.org/glass-history.html

[39] Glassforeurope [Internet] Main types of glass [updated 2013 May 23; cited 2014 Aug 17]. Available from: http://www.glassforeurope.com/en/products/main-types-of-glass.php

[40] Zhao YF, Liu Y, Jiang L, et al. A rare case of a glass fragment impacted in the parapharyngeal space associated with neurovascular compromise. Int J Oral Maxillofac Surg. 2011;40(2):209–211. PMID: 20817479, http://dx.doi.org/10.1016/j.ijom.2010.07.009 20817479 10.1016/j.ijom.2010.07.009 PMC3000444

[41] Cole C, Tuft S. Penetrating eye injury from rear view mirrors. Br J Ophthalmol. 2004;88(7):969–970. PMID: 15205250, http://dx.doi.org/10.1136/bjo.2003.038653 15205250 10.1136/bjo.2003.038653 PMC1772233

[42] Ozturk K, Keles B, Cenik Z, Yaman H. Penetrating zone ii neck injury by broken windshield. Int Wound J. 2006;3(1):63–66. PMID: 16650212. 16650212 10.1111/j.1742-4801.2006.00177.x PMC7951402

[43] Parry CD, Dewing S. A public health approach to addressing alcohol related crime in South Africa. Afr J Drug Alcohol Stud. 2006;5(1):41–56.

[44] Shindel AW, Rollins M, Dillon PA, Austin PF. Bladder injury from a shard of glass. J Trauma. 2006;61(6):1557 PMID: 17159710, http://dx.doi.org/10.1097/01.ta.0000235491.73456.f8 17159710 10.1097/01.ta.0000235491.73456.f8

[45] Baghai P, Sheptak PE. Penetrating spinal injury by a glass fragment: Case report and review. Neurosurgery. 1982;11(3):419–422. PMID: 7133359, http://dx.doi.org/10.1097/00006123-198209000-00014 7133359 10.1227/00006123-198209000-00014

[46] Dawson P, Goodwill AM. A review of weapon choice in violent and sexual crime. Sci Res. 2013;4(1):20–27. http://dx.doi.org/10.4236/blr.2013.41003

[47] Coomaraswamy KS, Shepherd JP. Predictors and severity of injury in assaults with bar glasses and bottles. Inj Prev. 2003;9(1):81–84. PMID: 12642566, http://dx.doi.org/10.1136/ip.9.1.81 12642566 10.1136/ip.9.1.81 PMC1730909

[48] Seggie J. Alcohol and South Africa's youth. S Afr Med J. 2012;102(7):587 PMID: 22748432. 22748432 10.7196/samj.6003

[49] Forcier MW. Unemployment and alcohol abuse: A review. J Occup Med. 1988;30(3):246–251. PMID: 3283302. 3283302

